# Opposite Interplay between PPAR Gamma and Canonical Wnt/Beta-Catenin Pathway in Amyotrophic Lateral Sclerosis

**DOI:** 10.3389/fneur.2016.00100

**Published:** 2016-06-28

**Authors:** Yves Lecarpentier, Alexandre Vallée

**Affiliations:** ^1^Centre de Recherche Clinique, Hôpital de Meaux, Meaux, France; ^2^CHU Amiens Picardie, Université Picardie Jules Verne, Amiens, France; ^3^Experimental and Clinical Neurosciences Laboratory, INSERM U1084, University of Poitiers, Poitiers, France

**Keywords:** amyotrophic lateral sclerosis, Wnt/beta-catenin, PPAR gamma, lithium, riluzole, bexarotene, tretinoin, retinoid acid

## Abstract

The opposite interplay between peroxisome proliferator-activated receptor gamma (PPAR gamma) and Wnt/beta-catenin signaling has led to the categorization of neurodegenerative diseases (NDs) as either NDs in which PPAR gamma is downregulated while the canonical Wnt/beta-catenin pathway is upregulated [amyotrophic lateral sclerosis (ALS), Parkinson’s disease, Huntington’s disease, multiple sclerosis, Friedreich’s ataxia] or NDs in which PPAR gamma is upregulated while the canonical Wnt/beta-catenin signaling is downregulated (bipolar disorder, schizophrenia, Alzheimer’s disease). ALS, a common adult-onset debilitating ND, is characterized by a chronic and progressive degeneration of upper and lower motor neurons resulting in muscular atrophy, paralysis, and ultimately death. The intent of this review is to provide an analysis of the integration of these two opposed systems, i.e., canonical Wnt/beta-catenin and PPAR gamma, in ALS. Understanding this integration may aid in the development of novel ALS therapies. Although the canonical Wnt/beta-catenin pathway is upregulated in ALS, riluzole, an enhancer of the canonical Wnt signaling, is classically prescribed in this disease in humans. However, studies carried out on ALS transgenic mice have shown beneficial effects after treatment by PPAR gamma agonists partly due to their anti-inflammatory effects.

## Introduction

Neurodegenerative diseases (NDs) are frequent and often present a bad prognosis. Both peroxisome proliferator-activated receptor (PPAR) gamma and the canonical Wnt/beta-catenin pathway play a key role in the pathophysiology of several NDs. The opposite interaction between PPAR gamma and the canonical Wnt/beta-catenin pathway has been reported in numerous studies (1–7). Certain NDs can be divided into two categories (8). On the one hand, there are NDs in which PPAR gamma is upregulated while the Wnt/beta-catenin pathway is downregulated, such as in bipolar disorder, schizophrenia, and Alzheimer’s disease. On the other hand, there are NDs in which PPAR gamma is downregulated while the Wnt/beta-catenin pathway is upregulated, such as in amyotrophic lateral sclerosis (ALS), Huntington’s disease, Parkinson’s disease, multiple sclerosis, and Friedreich’s ataxia. PPAR gamma agonists could exert protective effects in several NDs. They induce beneficial effects in ALS neurons of transgenic mice (9–12). Moreover, PPAR gamma coactivator-1alpha (PGC-1alpha) protects neurons and slows down the disease progression in ALS double transgenic mice (13, 14). PPAR gamma signaling may represent a therapeutic target in human ALS. Although the canonical Wnt/beta-catenin pathway is upregulated in ALS (15–18), riluzole, an enhancer of this system (19) represents a classically prescribed treatment of this disease in humans.

## Amyotrophic Lateral Sclerosis

Amyotrophic lateral sclerosis has been first described by J. M. Charcot in 1869. ALS, a fatal neurodegenerative disorder, is characterized by a chronic and progressive degeneration of the upper and lower motor neurons. This results in muscular atrophy, paralysis, and ultimately death. ALS is a common adult-onset debilitating ND. Its prevalence is about 5 per 100,000 individuals. Today, the pathophysiology of ALS in humans is not fully elucidated and is particularly complex partly due to various interconnected pathological mechanisms (20). 82% of ALS is sporadic. The most frequent mutations in familial ALS (FALS) or inherited are found in the SOD1 gene (Cu, Zn superoxide dismutase). FALS presents glutamate toxicity, mitochondrial dysfunction, and axonal transport defects (20). Many animal studies have used the mutant SOD1 transgenic mice (21). This model is reminiscent of the human ALS phenotype by developing a progressive motor neuron degeneration (22). The human sporadic ALS differs little clinically from SOD1-related FALS. Both forms induce motor neuron degeneration, paralysis, and death within 3–5 years from the appearance of the first symptoms. No pharmacological therapy can really stop the progression of ALS. Nevertheless riluzole is approved for the treatment of ALS patients although the benefits of this drug remain marginal (23–26).

## PPAR Gamma

Peroxisome proliferator-activated receptor gamma, a ligand-activated transcriptional factor, belongs to the nuclear hormone receptor superfamily. All PPARs heterodimerize with the retinoid X receptor (RXR), and they bind to peroxisome proliferator response elements, which are specific regions on the DNA of target genes. Naturally occurring agents directly activate PPAR gamma. PPAR gamma is expressed in various cell types, such as adipose tissue, immune cells, and brain cells, including astrocytes and microglia. This contributes to the anti-inflammatory response in the central nervous system (CNS) (27, 28). PPAR gamma regulates the expression of numerous genes implied in various cellular mechanisms, such as regulation of glucose homeostasis, insulin sensitivity, lipid metabolism, immune response, inflammation, and cell fate (29, 30). PPAR gamma is involved in several pathological states, such as diabetes, obesity, atherosclerosis, and cancer. PPAR gamma agonists, thiazolidinediones (TZDs), are insulin-sensitizing drugs, and certain TZDs have been used for treating type 2 diabetes. PPAR gamma diminishes inflammatory processes in many tissues. Moreover, PPAR gamma is a peripheral regulator of cardiovascular rhythms and controls circadian variations in blood pressure and heart rate through BMAL1 (31, 32). The role of PPAR gamma in neurodegeneration is well established (33). PPAR gamma agonists have been shown to be beneficial in several NDs, such as stroke, Alzheimer’s disease, ALS, Parkinson’s disease, Huntington’s disease, multiple sclerosis. PPAR gamma induces both neuroprotective and anti-inflammatory effects (27, 28). Astrocytic GLT1/EAAT2 gene, a target of PPAR gamma, induces neuroprotection by increasing glutamate uptake (34).

## Canonical Wnt/Beta-Catenin Pathway

**Figure 1:**
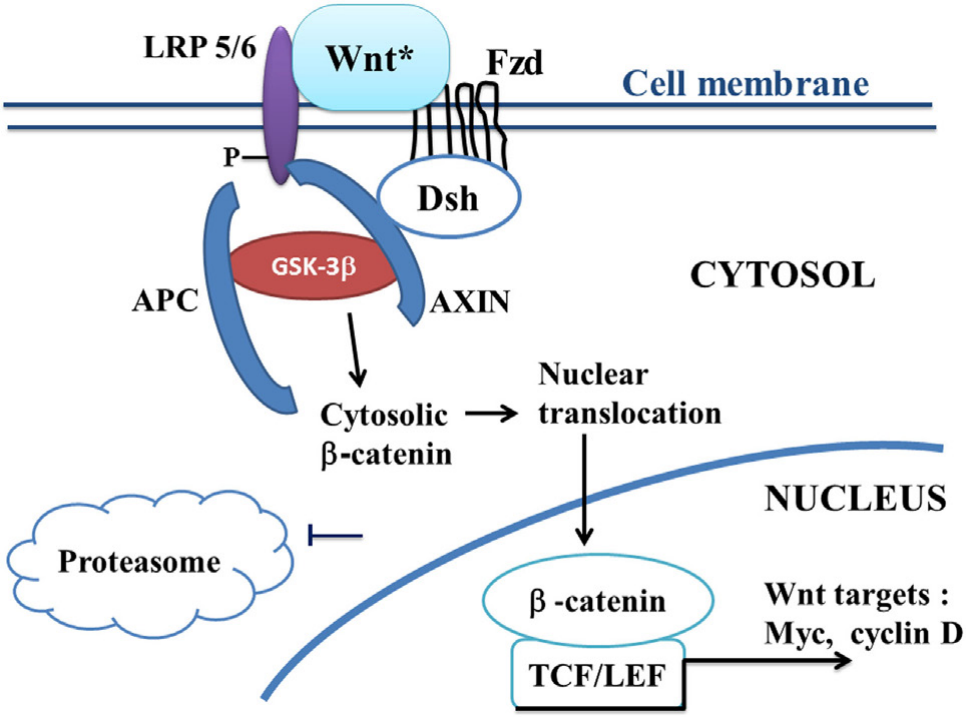
**A schematic model of the canonical Wnt/beta-catenin pathway in ALS**. In ALS, the canonical Wnt/beta-catenin pathway is upregulated (“on state”). Binding of Wnt to Fzd leads to activation of Dsh, which recruits the destruction complex to the plasma membrane. AXIN binds to the cytoplasmic tail of LRP5/6. Wnt also binds LRP5/6. This initiates LRP phosphorylation and Dsh-mediated Frizzled internalization. Activation of Dsh leads to the inhibition of GSK-3beta, which further reduces the phosphorylation and degradation of beta-catenin. The beta-catenin degradation complex AXIN/APC/GSK-3beta is inactivated with the recruitment of AXIN to the plasma membrane. Beta-catenin phosphorylation is inhibited. Then, beta-catenin accumulates into the cytosol and translocates to the nucleus to bind TCF–LEF co-transcription factors. This induces the canonical Wnt-response gene transcription (c-Myc, cyclin D, etc.). Abbreviations: APC, adenomatous polyposis coli; Dsh, Dishevelled; Fzd, Frizzled; GSK-3beta, glycogen synthase kinase-3beta; LRP5/6, low density lipoprotein receptor-related protein 5/6; TCF/LEF, T-cell factor/lymphoid enhancer factor; Wnt target genes: c-Myc, cyclin D; Wnt*, Wnt with ligands.

**Figure 2:**
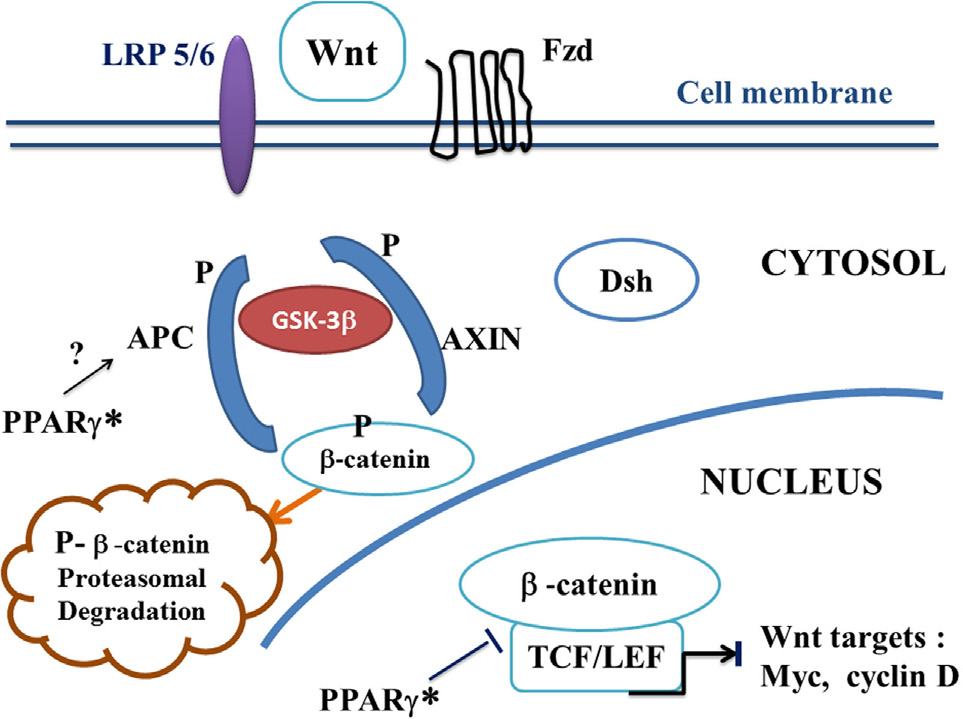
**A potential canonical Wnt/beta-catenin pathway in ALS treated by PPAR gamma agonists**. In the presence of PPAR gamma agonists, the “on state” of canonical Wnt/beta-catenin pathway may be interrupted at two potential levels. Firstly, *via* a hypothetical action of PPAR gamma* on APC. Inactivation of the destruction complex AXIN/APC/GSK-3beta is suppressed. Beta-catenin is phosphorylated by GSK-3beta. Thus, APC and AXIN complex with GSK-3beta and beta-catenin to enhance the destruction process of beta-catenin into the proteasome. Phosphorylated beta-catenin is recognized by an ubiquitin ligase, ubiquinated and degraded into the proteasome. The canonical Wnt pathway is in “off state.” Second, PPAR gamma can inhibit the beta-catenin/TCF/LEF complex into the nucleus, thus inhibiting the transcription of canonical Wnt target genes. Abbreviations: APC, adenomatous polyposis coli; Dsh, Dishevelled; Fzd, Frizzled; GSK-3beta, glycogen synthase kinase-3beta; LRP5/6, low density lipoprotein receptor-related protein 5/6; PPAR gamma*, PPAR gamma activated by agonists; TCF/LEF, T-cell factor/lymphoid enhancer factor; canonical Wnt target genes: c-Myc, cyclin D, etc.

Canonical Wnt/beta-catenin signaling plays a key role in embryonic development, cell migration, cell fate, and carcinogenesis (35, 36). Activation of the canonical Wnt pathway by Wnt ligands leads to increase the cytoplasmic beta-catenin level. Beta-catenin subsequently translocates to the nucleus and activates beta-catenin-specific gene transcription (1, 37) (Figure 1). In the absence of Wnt ligand, beta-catenin is recruited into the destruction complex that contains adenomatous polyposis coli (APC), Axin, and glycogen synthase kinase-3beta (GSK-3beta), which induces phosphorylation of beta-catenin, thus targeting it for ubiquitination and proteasomal degradation (Figure 2). In the presence of Wnt ligand, the binding of Wnt to Frizzled (Fzd) activates Dishevelled (Dsh). Dsh recruits Axin, which binds to the low density lipoprotein-receptor-related proteins (LRP5–6). Activation of Dsh inhibits GSK-3beta. This reduces phosphorylation and degradation of beta-catenin. The non-phosphorylated beta-catenin translocates to the nucleus and binds to T-cell/lymphoid-enhancing binding (TCF/LEF) transcription factors. This activates numerous target genes, including c-myc, cyclin D1, Axin-2, CD44, Cox2, and PPAR beta/delta (38, 39).

## Opposite Interplay Between PPAR Gamma and Canonical Wnt/Beta-Catenin Pathway

### PPAR Gamma Activation Induces Repression of the Beta-Catenin Pathway

Several studies have shown the opposite interaction between beta-catenin and PPAR gamma (1, 5, 40). The TZDs troglitazone, rosiglitazone, pioglitazone, and a non-TZD PPAR gamma activator GW1929 inhibit the transcription of beta-catenin (2, 3, 5, 6). Opposite interaction between PPAR gamma and beta-catenin involves the TCF/LEF factor-binding domain of beta-catenin and a catenin-binding domain of PPAR gamma (3). The antagonism between the beta-catenin pathway and PPAR gamma has been reported in various cell types, such as adipocytes (2) and hepatocytes (6). PPAR gamma suppresses the Wnt/beta-catenin pathway during adipogenesis (5). Conversely, activation of the canonical Wnt/beta-catenin pathway inhibits PPAR gamma expression (7).

### Deactivation of the Wnt/Beta-Catenin Pathway Induces Activation of PPAR Gamma

Inhibition of Wnt/beta-catenin pathway leads to increase the transcription of PPAR gamma (1). In preadipocytes, prevention of the Wnt pathway by overexpression of Axin induces differentiation into adipocytes. The canonical Wnt/beta-catenin/PPAR gamma system regulates the molecular switching of adipogenesis versus osteablastogenesis. The adipogenic pathway implies inhibition of Wnt signaling, leading to degradation of beta-catenin. This results in transcription of PPAR gamma, which initiates adipogenesis (7). Deactivation of the canonical Wnt/beta-catenin pathway and activation of PPAR gamma are also observed in arrhythmogenic right ventricular cardiomyopathy (ARVC) (1, 41). The suppression of canonical Wnt/beta-catenin signaling by plakoglobin (PG), i.e., gamma-catenin, recapitulates the phenotype of ARVC (1). The desmosomal PG has structural and functional similarities to beta-catenin (42). Gamma-catenin competes with beta-catenin through TCF/LEF transcription factors (43, 44). After competition with beta-catenin, gamma-catenin inhibits the canonical Wnt/beta-catenin-TCF/LEF pathway. This leads to enhance adipogenesis and fibrogenesis, as it is observed in human ARVC (1, 41, 45).

## ALS and PPAR Gamma

Neuroinflammation is commonly reported in NDs and particularly in ALS. PPAR gamma is a regulator of neuroinflammation and possibly represents a target for new therapeutic strategies. Beneficial effects induced by PPAR gamma agonists, partly due to their anti-inflammatory effects, have been observed in NDs. PPAR gamma inhibits NF-kappa B-mediated inflammatory signaling (46, 47). In ALS transgenic mice, beneficial effects of pioglitazone have been mainly ascribed to its anti-inflammatory activity. PPAR gamma agonists reduce neuropathological damages caused by inflammation in ALS (11). The involvement of PPAR gamma in ALS progression has been demonstrated in hSOD1^G93A^ mice using pioglitazone. Pioglitazone minimizes ALS-like symptoms (12) and increases survival in ALS transgenic mouse model (11). Pioglitazone-treated SOD1-G93A transgenic mice exhibit a delayed onset of the disease and survive significantly longer than non-treated transgenic mice. Pioglitazone induces neuroprotection in motor neurons of the spinal cord. The median fiber diameter of the quadriceps muscle is preserved. This indicates a morphological and functional protection of motor neurons due to pioglitazone (12). In neuron-specific PPAR gamma knockout mice and in response to middle cerebral artery occlusion, significant brain damages have been reported (48). Moreover, there is a reduced expression of certain genes, including SOD1 and glutathione S-transferase. Thus, in normal neurons, PPAR gamma is expressed and plays an important protective role. In addition to their anti-inflammatory effects, PPAR gamma agonists appear to be useful in neuroprotection. PPAR gamma activation induces neuroprotective effects in a *Drosophila* model of ALS, which recapitulates several aspects of the ALS phenotype (10). In a phase II double-blind controlled clinical trial, pioglitazone, in combination with riluzole, does not increase survival in ALS patients (49). The absence of beneficial effects in this trial might be partly due to the fact that riluzole activates the canonical Wnt/beta-catenin signaling in neuronal cells, as it will be discussed below (19). Moreover, pioglitazone is not a selective PPAR gamma agonist and belongs to the dual PPAR alpha/gamma agonists. Several studies have revealed a potential risk of bladder cancer in diabetic patients treated with pioglitazone, such as to call into question the benefit/risk ratio of this PPAR agonist (50). To highlight PPAR transcriptional activity, genetically engineered mice (PPRE-*Luc* mice) have been bred with the hSOD1^G93A^ALS mouse (51). In the PPRE-*Luc*^±^ *hSOD1^G93A^*^±^ double transgenic mice, PPAR gamma transcriptional activity increases in the spinal cord (9). PPAR gamma controls natural protective mechanisms against lipid peroxidation. This effect is correlated with the upregulation of lipid detoxification enzymes, such as glutathione S-transferase and alpha-2 lipoprotein lipase. These enzymes are implied in scavenging lipid peroxidation by-products.

## ALS and PPAR Gamma Coactivator-1 Alpha

Peroxisome proliferator-activated receptor gamma coactivator-1alpha, a transcriptional coactivator working together with PPAR gamma, plays an important role in numerous NDs (33). Increase in expression of PGC-1alpha in transgenic SOD1-G93A mice prevents neuronal cell death (52). SOD1-G93A mice present mitochondrial abnormalities in motor neurons of the spinal cord, and PGC-1alpha induces neuroprotection. Expression of PGC-1alpha reduces motor neuron degeneration in SOD1-G93A transgenic mice (13, 14). In PGC-1alpha transgenic mice crossed with SOD1 mutant G93A mice, there is a neuroprotection, and the progression of the disease is significantly slowed (13). Motor function and survival are improved by using a double transgenic mouse model where PGC-1alpha is overexpressed in a SOD1 transgenic mouse (14). In this double transgenic mouse model, there are decreased weight loss, improved motor performance, slowed disease progression, and reduced motor neuronal death. In a mouse model of inherited ALS, increased PGC-1alpha activity sustains mitochondrial biogenesis and muscle function without extending survival (53).

## ALS, PPAR Gamma, Retinoid X Receptor, and Retinoic Acid

Peroxisome proliferator-activated receptor and RXR receptor form heterodimers to become functional. There is a convergence of the 9-cis retinoic acid and PPAR pathways through heterodimer formation of the two receptors (54). Retinoid signaling is important in the development of the CNS and contributes to regenerative processes that occur after injury. In ALS, the retinoid signaling is involved in gene expression in the spinal cord (55). Abnormalities of the anaplastic lymphoma kinase (ALK) gene are associated with sporadic ALS. Reduced ALK mRNA levels have been reported in ALS patients (56). Midkine (MK), a ligand for ALK, is decreased in patients with sporadic ALS (57). MK expression is induced by the retinoic acid signaling. Moreover, MK activates ALK. Retinoic acid gene improves survival in the ALS SOD1-G93A transgenic mice (58). In ALS patients treated with tretinoin (i.e., retinoic acid), pioglitazone combined with riluzole has demonstrated no slowing on the disease progression (59). This might be partly explained by the fact that riluzole is an enhancer of the canonical Wnt pathway, as reported below. Bexarotene (Bxt), a highly selective RXR agonist, induces neuroprotective effect in the SOD1^G93A^ mouse model of ALS (60). Bxt significantly delays the early motor neuron degeneration in ALS transgenic mice and partially preserves the spinal motor neuron loss. Bxt ameliorates the loss of body weight and increases mouse survival up to 30% of the symptomatic period. Bxt preserves the neuromuscular function.

## ALS and Canonical Wnt/Beta-Catenin Pathway

The canonical Wnt/beta-catenin signaling is involved in numerous NDs, particularly in ALS. Many studies have shown that this pathway is upregulated in motor neurons of ALS mouse model (15–18). In the spinal cord of SOD1-G93A ALS transgenic mice, mRNAs and proteins of both Wnt2 and Wnt7a are upregulated (16). Activation of the Wnt signaling inhibits the GSK-3beta activity. In the spinal cord of these mice, mRNAs and proteins of both Wnt3a and Cyclin D1 are upregulated (15). Beta-catenin translocates to the nucleus and subsequently activates transcription of the target gene Cyclin D1. Increase in Wnt5a and Fzd2 expression in the spinal cord of SOD1-G93A ALS transgenic mice, SOD1-G93A transfected NSC-34 cells, and primary cultures of astrocytes from SOD1-G93A transgenic mice have been shown to be changed (17). In the spinal cords of SOD1-G93A ALS transgenic mice, expression of Wnt1 and Fzd1 is increased (18).

## ALS, Riluzole, and Lithium

No really efficient treatment exists for ALS (61, 62). However, riluzole is approved for the treatment of ALS in most countries. This is based on the results supporting a role of glutamate toxicity in ALS. Riluzole has numerous pharmacological properties, i.e., presynaptic inhibition of the glutamate release, inhibition of G-protein-dependent processes, blockade of the voltage-gated sodium channel, and modulation of *N*-methyl-d-aspartate ionotropic receptor (61). Two trials (23, 24) have shown a prolongation of median survival by 2–3 months by using riluzole in human ALS. Two other studies have led to similar conclusions (25, 26). Interestingly, riluzole has been shown to be an enhancer of the Wnt/β-catenin signaling in melanoma (19). Treating melanoma cells with riluzole *in vitro* enhances the ability of Wnt3A to regulate gene expression and decrease cell proliferation. Importantly, riluzole also enhances the Wnt/beta-catenin pathway in both HT22 neuronal cells and adult hippocampal progenitor cells (19). As the Wnt/beta-catenin pathway is upregulated, at least in genetic ALS mice (15–18), this might partly explain the poor results reported in trials testing riluzole in ALS (23–26). The GSK-3beta-inhibitor lithium, an activator of the canonical Wnt/beta-catenin signaling (63–65), has also been evaluated as a treatment for ALS (66). Surprisingly, in ALS patients treated with lithium, the disease progression appears to be markedly attenuated. In a parallel study on the genetic SOD1-G93A mouse ALS animal model, lithium induces a marked neuroprotection, delaying disease onset and duration and increasing the life span. Lithium is logically used for the treatment of bipolar disorder in which the Wnt/beta-catenin signaling is downregulated (67, 68). Thus, riluzole reduces symptoms of obsessive–compulsive disorder, unipolar and bipolar depression, and generalized anxiety disorder (69). This is expected because the Wnt/beta-catenin pathway is downregulated in bipolar syndrome and because riluzole, like lithium, are enhancers of the Wnt/β-catenin signaling (19).

## Conclusion

Peroxisome proliferator-activated receptor gamma agonists represent promising therapeutics for certain NDs, such as ALS. PPAR gamma agonists appear to be beneficial in several ALS transgenic animal models partly due to their anti-inflammatory properties. Moreover, PPAR gamma activation induces repression of the beta-catenin pathway (2, 3, 5, 6). This represents the rationale for the use of PPAR gamma agonists in case of downregulation of PPAR gamma and upregulation of the Wnt/beta-catenin pathway, such as in ALS. New PPAR gamma agonists, specific and devoid of adverse side effects, might represent a new therapeutic approach for the treatment of ASL. PPAR gamma agonists increase survival in ALS transgenic mice and minimize neuroinflammation. Rather than stimulation of the canonical Wnt/beta-catenin pathway by riluzole administration, inhibition of this system might also represent a therapeutic approach in ALS.

## Author Contributions

The authors YL and AV contributed equally to this mini review.

## Conflict of Interest Statement

The authors declare that the research was conducted in the absence of any commercial or financial relationships that could be construed as a potential conflict of interest.
